# Case Report: Caecal volvulus management from diagnosis to treatment in a young patient

**DOI:** 10.12688/f1000research.121789.1

**Published:** 2022-07-12

**Authors:** Imed Abbassi, Wissem Triki, Racem Trigui, Ahmed Itaimi, Karim Ayed, Hajer Sebri, Oussema Baraket, Sami Bouchoucha

**Affiliations:** 1 General Surgery Department, Hôpital Universitaire Bougatfa of Bizerte, Bizerte, 7000, Tunisia; 2 Gynecology and Obstetric Department, Hospital Mongi Slim of La Marsa, Tunis, 1000, Tunisia

**Keywords:** caecal volvulus, whirl sign, caecopexy, caecectomy

## Abstract

Caecal volvulus (CV) is a rare cause of intestinal obstruction, defined by an axial torsion of the caecum, ascending colon, and terminal ileum around the mesenteric vascular pedicles, leading to ischemia and bowel necrosis.

A 20-year-old woman, with no significant medical history, was admitted for generalized abdominal pain evolving for three days, along with constipation and abdominal distension, but with no vomiting. Physical examination showed a generalized abdominal tenderness with no rigidity or rebound tenderness, associated with abdominal distension and tympanic upon percussion. Laboratory findings were within normal limits.

An abdominal computed tomography scan revealed distension of a loop of the large bowel with its long axis extending from the right lower quadrant to the epigastrium or left upper quadrant. Colonic haustral pattern was absent. An abdominal computed tomography scan showed a rounded focal collection of air-distended bowel with haustral creases in the upper left quadrant. In addition, spiraled loops of the collapsed cecum (giving a whirl sign) were noted, along with low-attenuating fatty mesentery from the twisted bowel.

The patient underwent an emergency laparotomy and caecectomy using GEA 80 charges. The patient had no complaints post-operation.

CV is a rare cause of bowel obstruction, mainly caused by an exceedingly mobile caecum. Despite its rareness, CV represents the second most common cause of large bowel volvulus, behind sigmoid volvulus. For acute obstruction by CV, it is hard to differentiate it clinically from obstruction of the small bowel; therefore, radiological exams are needed. Surgery is the gold standard treatment for CV.

We report a rare case of CV to highlight the rarity of this pathology, specify its diagnostic and therapeutic means, and its clinical and biological evolution.

## Introduction

Volvulus is commonly defined as a twisted loop of the intestinal bowel and associated mesentery around a fixed point at its base. Caecal volvulus (CV) is a rare cause of intestinal obstruction, defined by an axial torsion of the caecum, ascending colon, and terminal ileum around the mesenteric vascular pedicles. ^1^

Preoperative diagnosis can be difficult due to its unspecific symptoms. As a result, CV is a surgical emergency and any delay in management can be associated with complications mainly bowel ischemia eventually leading to perforation and peritonitis.

We report this case of CV to highlight the rarity of this pathology, the difficulty of clinical diagnosis, and to report the success of caecostomy as a surgical option against right colectomy.

## Case presentation

A 20-year-old Tunisian woman, unemployed, with no significant medical history, was admitted for generalized abdominal pain evolving for three days, along with constipation and abdominal distension, but with no vomiting. Physical examination showed a generalized abdominal tenderness with no rigidity or rebound tenderness, associated with abdominal distension and tympanic upon percussion. Laboratory findings were within normal limits.

**Figure 1.**
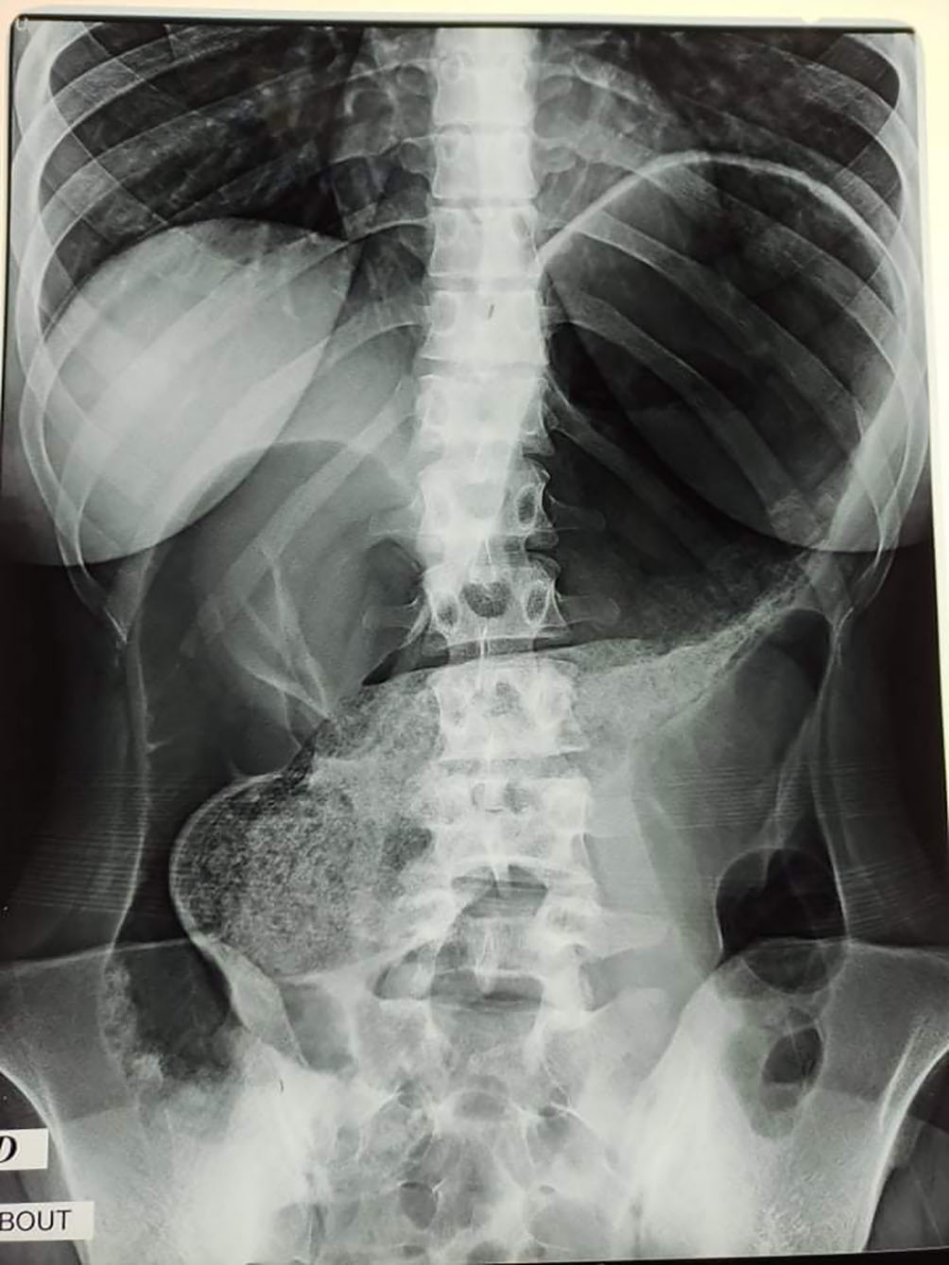
Abdominal X-ray showing the Significant distension of the colon reaching the epigastrium and the left hypochondrium.

Abdominal X-rays were taken, and they revealed distension of a loop of the large bowel, with its long axis extending from the right lower quadrant to the epigastrium or left upper quadrant. Colonic haustral pattern was absent ( Figure 1).

**Figure 2.**
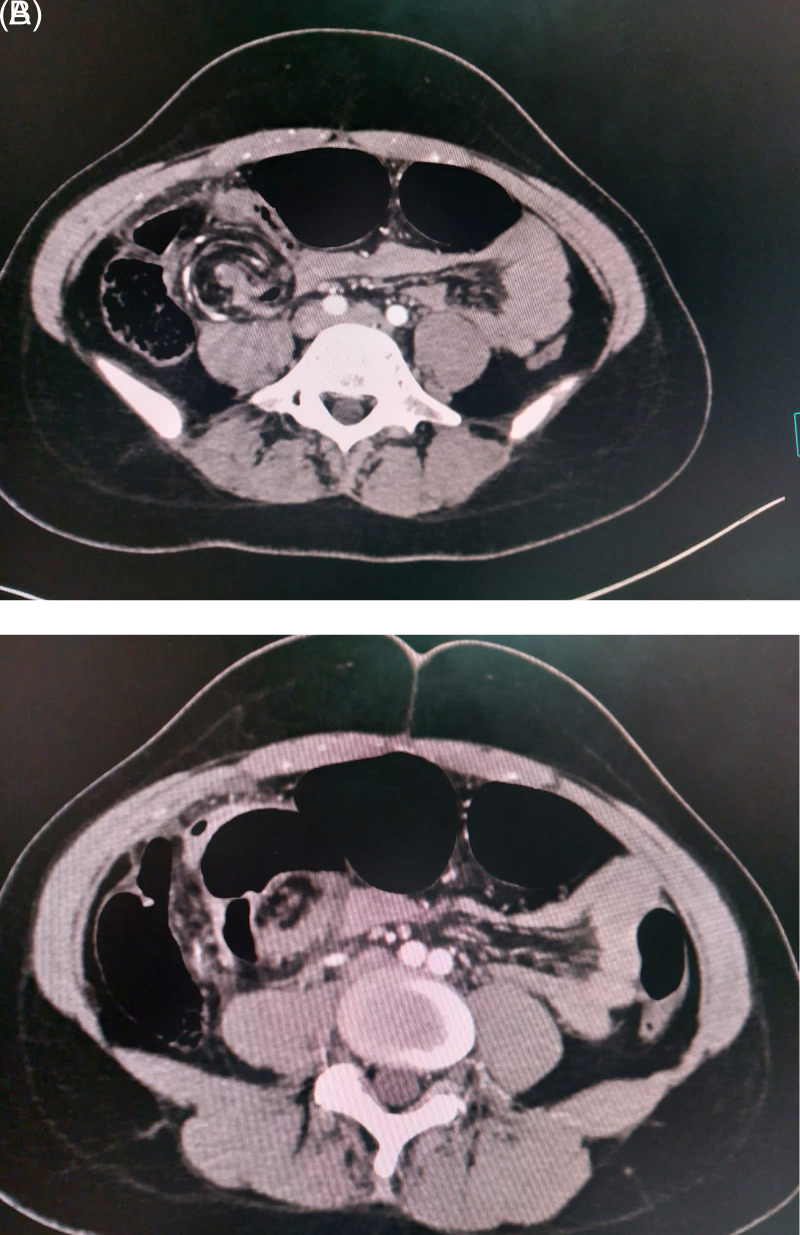
Major caecal distension related to a caecal volvulus responsible for parietal thinning and a fatty mesentery with low attenuation after contrast injection.

An abdominal computed tomography (CT) scan showed a rounded focal collection of air-distended bowel with haustral creases in the upper left quadrant. In addition, spiralled loops of collapsed cecum (giving a whirl sign) were noted, with low-attenuating fatty mesentery from the twisted bowel ( Figure 2).

The patient was kept for six hours under observation, with nothing by mouth, nasogastric tube suction, and IV fluids. At the end of the evaluation period (six hours post suction), the patient underwent an emergency laparotomy.

**Figure 3.**
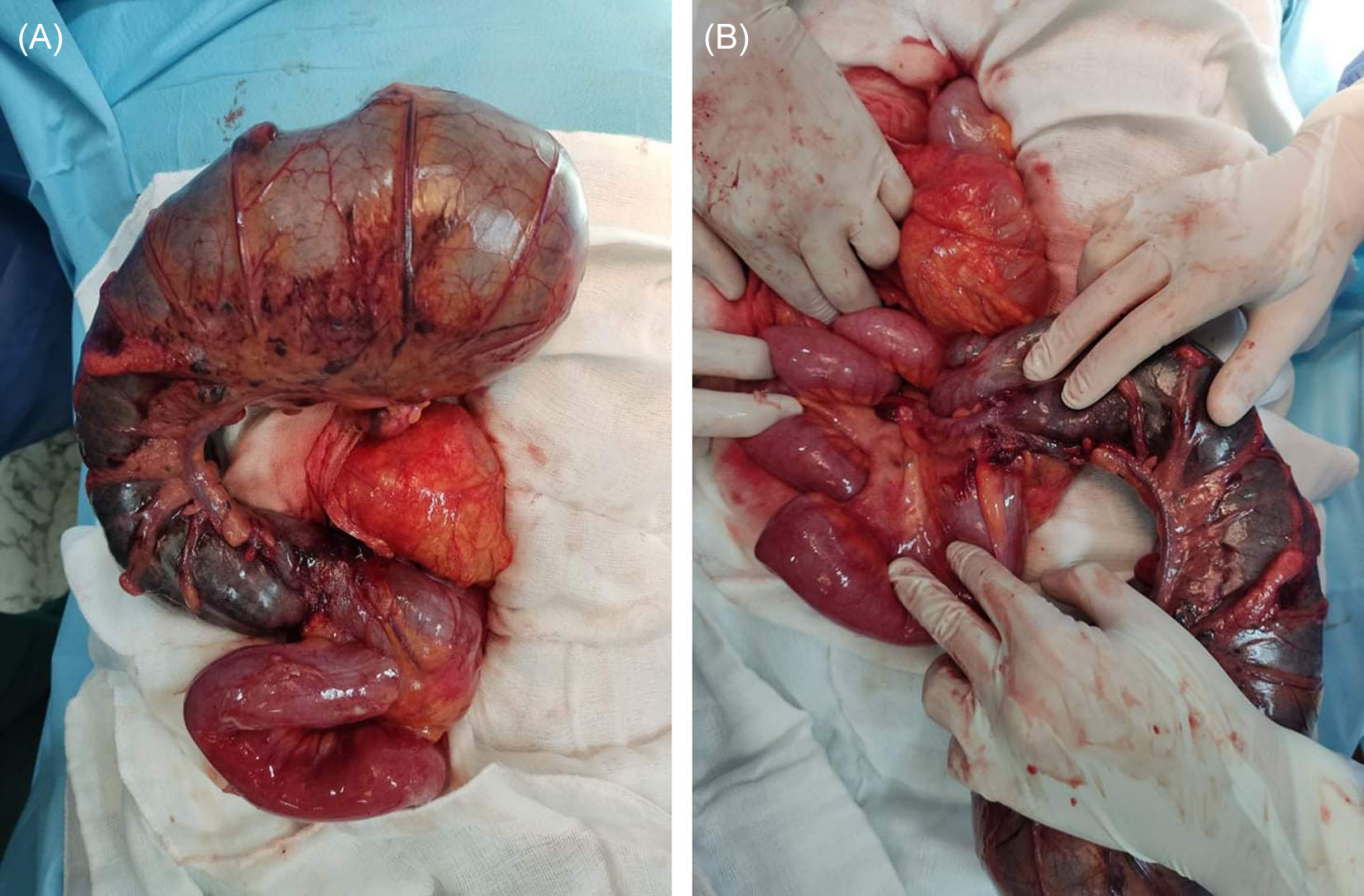
Severely distended cecum occupying the right hemi-abdomen with a vertex extending into the left hypochondrium associated with necrosis and pre-perforative signs.

During the operation, the caecum in the left hypochondrium was hugely distended and contained signs of pre-perforation, such as bowel deperitonization lesions ( Figure 3). CV was seen, and clockwise de-rotation of volvulus was performed. After CV was diagnosed, a caecectomy was performed using GEA 80 charges.

The patient had an adequate postoperative evolution. Antibiotics prophylaxis was given for 48 hours post-operatively with amoxicillin (Ac. Clavulanique intravenously at the dosage of 1gr three times a day). Venous thromboembolism prophylaxis was given using enoxaparin subcutaneously at a dosage of 4000 UI once a day. A liquid diet was initiated 48 h after surgery. The patient had no clinical signs of leakage. On day 5, the patient was discharged. After two weeks, the final histopathological examination was performed, reporting caecal necrosis without underlying lesions.

In the postoperative assessment after two months, the patient was found to be tolerating oral intake, was asymptomatic, and did not have any signs of weight loss.

## Discussion

CV is an infrequent cause of colon obstruction. ^2^ It is the second most frequent location of colonic volvulus and accounts for up to 60% of cases according to several studies. ^3^ To be diagnosed with CV, two conditions must be met: an abnormal mobile caecum associated with the lack of attachment of the mesenterium, caecum, or right colon to the posterior peritoneum ^3^^–^^5^; and a fixed point around which the caecum can twist. ^6^

A mobile caecum is an anatomical variant, present in 25% of the general population according to some studies. ^7^ It is believed that a mobile caecum is caused by deficient colonic fixation to the peritoneum or colon elongation resulting from over-rotation during embryologic development. ^8^ Clinical presentation is exceedingly variable, but the most common symptoms are abdominal, accompanied by nausea, vomiting, and abdominal distension. ^9^

In general, CV can be presented in three clinical syndromes: recurring intermittent pain, acute abdominal obstruction, and devastating acute obstruction. ^2^ In recurring intermittent volvulus, patients experience pain in their lower right quadrant associated with abdominal dilation partially relived by gas release, such as the case of our patient. ^10^^,^^11^ For acute obstruction by caecal volvulus, it is hard to differentiate it clinically from obstruction of the small bowel; therefore, radiological exams are needed. ^3^ If not treated on time, an acute obstruction may progress into a devastating acute obstruction associated with severe abdominal pain, sepsis, and peritoneal irritation caused by necrosis and intestinal perforation due to obstruction and twisted mesenteric vessels. ^12^^,^^13^

In terms of diagnosis, CT is the imaging technique of choice, allowing not only confirmation of the diagnosis, but also ruling out other causes of acute obstruction. Coffee bean, bride beak, and whirl signs are the most common observations identified during CT. ^14^

Surgery is the main course of treatment for CV, ranging from simple detorsion to right colectomy. ^7^ If gangrene, necrosis, or perforation are identified, resection is mandatory, and the current method of choice is right colectomy with primary anastomosis or ileostomy depending on perioperative factors. ^12^ Three main procedures are described in the literature following caecum detorsion with no suspicion regarding its viability: isolated detorsion, caecopexy, and caecostomy. ^8^ Isolated detorsion without caecopexy or cecostomy is associated with a high risk of recurrence; therefore, it should not be used anymore. ^15^ Caecopexy is obtained by attaching the right colon to the parietal peritoneum with a recurrence rate of up to 40%. Cecostomy is associated with a higher risk of complications, including caecum gangrene, fistula, and leakage. In this case, our patient was young with signs of caecal pre perforation and not prepared for a right colectomy; as a result, we choose to perform a caecostomy. Compared to caecopexy, caecostomy has a higher rate of morbidity and mortality. As a result, caecopexy is recommended for patients with viable intestines not tolerating right colectomy and those suffering from mobile caecum syndrome. ^8^^,^^11^^,^^16^ This, in conjunction with modern-day laparoscopic evolution, has decreased mortality, and post-operative results have markedly improved. ^3^

## Conclusion

Caecal volvulus is a rare cause of bowel obstruction, mainly caused by an exceedingly mobile caecum. Early diagnosis can be difficult due to its unspecific symptoms. Computed tomography plays a major role in a positive diagnosis. The main course of treatment is surgical, and modalities depend on various factors such as patient status and perioperative findings. Nowadays laparoscopic evolution continues to reduce postoperative morbidity.

## Consent

Written informed consent to publish this case report and any associated images was obtained from the patient.

## Data availability

All data underlying the results are available as part of the article and no additional source data are required.
