# Supplementary material for: Case Report: Caecal volvulus management from diagnosis to treatment in a young patient
Source: F1000Res. 2022 Nov 2;11:781. Originally published 2022 Jul 12. [Version 2] doi: 10.12688/f1000research.121789.2 (PMC9360906; doi:10.12688/f1000research.121789.2)
Supplement: Peer review history

# Peer review: Reviewer response for version 2

**DOI:** 10.5256/f1000research.139079.r154838

**Published:** 2022-11-07

**Authors:** DANIEL WAISBERG

**Affiliations:** 1 Liver and Gastrointestinal Transplant Division, Department of Gastroenterology, Faculty of Medicine, Universidade de São Paulo, São Paulo, Brazil

The authors did not respond to the issues raised in the previous review nor did they implement any changes in the manuscript. I suggest authors to read my previous comments in order to improve their manuscript.

Are enough details provided of any physical examination and diagnostic tests, treatment given and outcomes?

Partly

Is the case presented with sufficient detail to be useful for other practitioners?

Yes

Is sufficient discussion included of the importance of the findings and their relevance to future understanding of disease processes, diagnosis or treatment?

Partly

Is the background of the case’s history and progression described in sufficient detail?

Yes

Reviewer Expertise:

Gastrointestinal Surgery

I confirm that I have read this submission and believe that I have an appropriate level of expertise to confirm that it is of an acceptable scientific standard, however I have significant reservations, as outlined above.

# Peer review: Reviewer response for version 1

**DOI:** 10.5256/f1000research.133688.r146450

**Published:** 2022-08-19

**Authors:** DANIEL WAISBERG

**Affiliations:** 1 Liver and Gastrointestinal Transplant Division, Department of Gastroenterology, Faculty of Medicine, Universidade de São Paulo, São Paulo, Brazil

**Comments to the Authors**

 Abbassi et al performed an interesting case report of caecal volvulus. Some issues with this manuscript are detailed below.

1. **Abstract**
  - The findings of the abdominal X-rays are described as they were from the abdominal CT scan
  - The correct term is “GIA 80 stapler” instead of “GEA 80 charges”
2. **Introduction **
  - It is mentioned that the objective of this report is to address the success of caecostomy, but in the case presentation it is stated that the patient underwent caecectomy (resection). Which treatment was perfomed? Please clarify
  - We suggest using the sentence: “We report A case of (…)
3. **Case Presentation**
  - We suggest using the term: “fasted” instead of “nothing by mouth”
  - The correct term is “GIA 80 stapler” instead of “GEA 80 charges”
  - Please specify which treatment was performed: caecostomy or caecectomy (resection). If it was a resection, was primary anastomosis performed?
  - “Clavulanique” is a French word
  - If the diagnosis of CV was already made by abdominal CT scan, why was the patient kept under observation for 6 hours? Why was emergency laparotomy or laparoscopic not performed after diagnosis?
4. **Discussion **
  - The authors state that CV is an infrequent cause of colon obstruction, but then the mention it is responsible for 60% of colonic volvulus. Is this data correct?
  - We suggest using the sentence: “its use is not currently advocated” instead of “it should not be used anymore?
  - The authors stated they performed a caecostomy. Is this correct? Why not resect the caecum? Please clarify which surgery was performed and justify its choice in the discussion

Are enough details provided of any physical examination and diagnostic tests, treatment given and outcomes?

Partly

Is the case presented with sufficient detail to be useful for other practitioners?

Yes

Is sufficient discussion included of the importance of the findings and their relevance to future understanding of disease processes, diagnosis or treatment?

Partly

Is the background of the case’s history and progression described in sufficient detail?

Yes

Reviewer Expertise:

Gastrointestinal Surgery

I confirm that I have read this submission and believe that I have an appropriate level of expertise to confirm that it is of an acceptable scientific standard, however I have significant reservations, as outlined above.

# Peer review: Reviewer response for version 1

**DOI:** 10.5256/f1000research.133688.r146154

**Published:** 2022-08-08

**Authors:** Houcine Magherbi

**Affiliations:** 1 General Surgery Department, La Rabta Hospital, Tunis, Tunisia

This is an interesting paper which describes a case of cecal volvulus (CV).

 CV is an uncommon cause of colonic obstruction. Diagnosis is difficult and, if delayed, the results may be intestinal ischemia, perforation, sepsis, and even death.

 First-line treatment for cecal volvulus is surgery, as nonoperative management is rarely achievable.

 That Is why I want to ask the authors why they perform an evaluation period of six hours? They must insist that Caecum Volvulus requires immediate surgery because the delay in diagnosis is responsible of high rate of mortality. They should also mention if there was previous abdominal surgery, history of chronic colicky abdominal pain or intermittent bowel subocclusion.

 Please correct those sentences:

 “After CV was diagnosed, a caecectomy was performed using GEA 80 charges. -> After CV was diagnosed, a caecectomy was performed using GIA 80 surgical staplers”

 “A mobile caecum is an anatomical variant, present in 25% of the general population according to some studies."

 This is overestimated because mobility of the cecum and ascending colon has been estimated to occur in 10–20 % of population.

 You can mention that several authors have reported successful colonoscopic reduction of cecal volvulus. The manuscript is well described and all the other parts are well described with adequate diagnostic steps.

Are enough details provided of any physical examination and diagnostic tests, treatment given and outcomes?

Yes

Is the case presented with sufficient detail to be useful for other practitioners?

Yes

Is sufficient discussion included of the importance of the findings and their relevance to future understanding of disease processes, diagnosis or treatment?

Yes

Is the background of the case’s history and progression described in sufficient detail?

Yes

Reviewer Expertise:

surgery

I confirm that I have read this submission and believe that I have an appropriate level of expertise to confirm that it is of an acceptable scientific standard.

# Peer review: Reviewer response for version 1

**DOI:** 10.5256/f1000research.133688.r144137

**Published:** 2022-08-04

**Authors:** Sardar Hassan Arif

**Affiliations:** 1 Department of Surgery, College of Medicine, University of Duhok, Kurdistan Region, Iraq

This was a good case report written in a proper manner.

 Some few comments for the authors in the management of case:

1. Better to mention in the history that there was no history of previous abdominal operation.
2. Why was the case kept on observation for 6 hours while it is very clear case of intestinal obstruction, only resuscitation for 1-2 hours enough?
3. During the operation it is better to put clamps on proximal and distal before de-rotation to prevent endotoxins being liberated into circulation.

Are enough details provided of any physical examination and diagnostic tests, treatment given and outcomes?

Yes

Is the case presented with sufficient detail to be useful for other practitioners?

Yes

Is sufficient discussion included of the importance of the findings and their relevance to future understanding of disease processes, diagnosis or treatment?

Yes

Is the background of the case’s history and progression described in sufficient detail?

Yes

Reviewer Expertise:

NA

I confirm that I have read this submission and believe that I have an appropriate level of expertise to confirm that it is of an acceptable scientific standard.
